# COVID-19 – the ultimate disruptor?

**DOI:** 10.15694/mep.2020.000104.1

**Published:** 2020-05-20

**Authors:** Alexander Woywodt, Hetty Breed, Colin Lumsden

**Affiliations:** 1 Lancashire Teaching Hospitals NHS Foundation Trust; 2 Lancashire Teaching Hospitals NHS Foundation Trust / University of Manchester; 3 University of Aberdeen

**Keywords:** COVID-19, curriculum, telemedicine, professionalism

## Abstract

This article was migrated. The article was marked as recommended.

Coronavirus disease 2019 (COVID-19) has influenced undergraduate medical education in various ways already. In affected countries, educators and their teams were faced with a rapidly changing situation that made traditional ways of curriculum delivery impossible and required alternative approaches. Exams have also been affected and a cohort of students has graduated early and now joins the workforce. There is also concern for the next academic year should the pandemic last longer. In this paper we aim to describe wider implications of the pandemic beyond current curriculum delivery, exams and planning. We describe how our own clinical and educational environment has been utterly transformed within weeks and speculate how much these changes will persist after the pandemic. We also describe student concerns and introduce the thought that the pandemic may have positive long term effects as well. Finally, we speculate how COVID-19 may affect student recruitment, multi-professional learning and the current and future undergraduates’ view of the profession. Our aim is to share our experience in the UK, reflect on the direction and magnitude of change seen in our own local and regional practice, and provide food for thought for educators and their teams who find themselves in a similar situation.

## Introduction

An ever-increasing number of publications have described epidemiology, clinical features and treatment of Coronavirus disease 2019 (COVID-19). The educational literature has noted obvious implications, such as disruption of exams ( Boursicot *et al.*, 2020), an increased use of technology to deliver content ( Goh and Sandars, 2020) and the long-term effects of this trend ( Sabzwari, 2020). Here, we describe further significant effects of the pandemic on our own educational practice in the United Kingdom’s National Health Service (NHS). We contrast negative as well as positive effects on medical education and speculate about the long-term transformative power of COVID-19.

## Effects of the COVID-19 pandemic: Observation and reflection

Our own practice in medicine has seen a complete transformation within weeks whereby most of our consultations are now virtual and video-based. We appreciate that in some specialties, such as stroke medicine, or in rural or remote areas, telemedicine was already well established but in our institution it was virtually non-existent prior to now. For many of our patients observing government advice to stay at home, virtual meetings have also become the new norm in their own professional and social lives. The pandemic has now proven to them that the same technology also works for virtual consultations, that it is widely available and that it works well for most routine clinical appointments. It is difficult to predict how much this development will continue after the pandemic but based on our recent clinical encounters we believe that much of this development will be irreversible. We feel that most of our patients, and certainly younger patients and those with a significant commute to outpatient clinics, will be reluctant to revert to the traditional face to face model of outpatient medicine. Based on our experience in the last six weeks the crisis will therefore greatly accelerate the uptake of telemedicine in the medical specialties. The trend may spare some areas where hands-on examination is pivotal, such as surgery or smaller surgical specialties. This development is further compounded by the fact that some colleagues are now holding telephone or video clinics from the comfort of their own home. Taken together, a significant proportion of our learning opportunities for examination and history taking have simply disappeared within weeks, and with very little hope of return. It is already difficult to see how our future students could fulfil even the minimum requirements for clinical examination and history taking unless we maximise learning opportunities provided by inpatient care, possibly with the help of technology to identify suitable inpatients ( Singh *et al.*, 2020). We also need to consider aspects of equipment, for example where physicians conduct virtual consultations via headset/microphone: Without intervention and investment in suitable kit, our students will find it difficult to partake in such consultations, let alone learn how to carry out such tasks.

These observations add further to the more obvious negative effects of COVID-19 on medical education ( Table 1A). Of those concerns the practical implications can be addressed in some way. However we worry about the challenge of recruiting tutors from an exhausted workforce, which may also take the pandemic as an opportunity to reflect on their work-life balance and avoid optional educational roles. We are also concerned that the colossal levels of spending during the pandemic will lead to significant austerity later, with all its challenges for education ( Cartwright *et al.*, 2017). The student perspective on COVID-19 is also important and a number of significant concerns have been voiced ( Table 1B). One recurring theme is lack of time for current fifth year medical students to receive relevant teaching before entering practice as junior doctors. Neither will there be time to work out the logistics of students observing virtual or telephone clinics, and it is likely that current students will miss out on valuable learning experiences. With the huge loss of learning our current students have already faced, we must begin to think about the integration of technology into both pre-clinical and clinical experience and into exams to ensure the education of our future doctors is not compromised. We should probably also consider the resilience of our programmes overall and we note that the impact on our own programme could have been so much worse had the pandemic occurred at the start of the academic year and not in spring.

**Table 1.** A: Possible negative consequences of the COVID-19 pandemic on medical educationB: Concerns among UK medical students regarding the effects of COVID-19 on their education. Adapted from ( N.N., 2020).

A: Negative Consequences
1. Short term • Disruption of exams, graduation and planning for next academic year • Further loss of clinical learning if pandemic continues longer than anticipated • Loss of research opportunities due to project cancellations 2. Mid term • Recruiting tutors for the next academic year from a pool of exhausted clinicians • Disruption to start of the next academic year if social distancing rules continue • Cost and training implications of integrating telemedicine into education • Suitable hardware not available for students to participate 3. Long term • Loss of hands-on practical experience with examinations and clinical skills • Clinicians working from home with significant loss of learning opportunities • Workforce implications due to burn out, career change and retirement • Spending during the pandemic makes another wave of austerity extremely likely
**B: Student concerns**
• No time to de-stress from final exams • De-standardisation of final exams • Missing out on specialty placements • Worried about not being prepared for final year • Lack of information from Medical Schools • Why can some students progress without doing exams yet others must complete final year exams • May not get to work where we want • Lack of preparation to work with COVID-19 • People may look down on students who are not involved in volunteering • Lack of personal protective equipment and supervision • What is clinically expected of volunteers? • How will volunteering affect progression? • Impact of cancelled assessments on honours points and foundation programme applications • Implication of cancelled assessments on future applications to intercalate • What pastoral support will be made available to students volunteering during the pandemic

However, the effects of the pandemic may not be uniformly detrimental: One very positive development is how students have offered to help, either as volunteers or in newly defined roles devised by their medical schools and hospitals. The evolving healthcare emergency has seen an astounding response from those studying medicine as a career. The University of Aberdeen Medical School sought volunteers for an Interim Foundation Year Post (final year graduates) and those willing to support clinical areas from roles as diverse as health care support workers to cleaning and portering. The response has been very positive: 184 final year students were surveyed. 3 students did not respond. 145 (80%) of final year students volunteered to work in an interim Foundation pre-registration post. 29 (16%) were unsure although of those 13 were prepared to work as Health Care Support Workers (HCSW). 7 (4%) stated that they would not volunteer. In total 87% of final year students were prepared to volunteer with little concern for their personal safety while a further 9% were unsure. Of those not willing to volunteer 1 cited having just given birth, 1 cited travel home (overseas student) and 1 wanted a financial benefit before committing but would work as a health care support worker. 517 students from Years 1-4 of the MBChB programme volunteered and 316 from the BSc in Medical Science volunteered. In addition students have sought to link volunteering students with healthcare providers e.g. through the Medical Schools Council. The response to calls for volunteers seen by us and by others ( Rasmussen *et al.,* 2020) demonstrates clearly the vocational nature of medical training whereby those in education are willing to place themselves on the front line for the greater good of the community. Other positive effects from the pandemic are also conceivable ( Table 2).

**Table 2.** Possible positive consequences of the COVID-19 pandemic.

• Volunteering as a positive experience • Health care professionals highly regarded by the public • Public and visible support and appreciation of individual health care professionals and the health system in general • Seeing the health system cope and overcome the crisis • Experiencing leadership during the crisis • Witnessing effective communication during the crisis • Innovation and service development at unprecedented pace • Opportunities for teams and care systems to question, amend, or stop established pathways • Improvement in patient care that will last beyond the pandemic • Opportunities for inter-professional learning • Breakdown of traditional hierarchical structures • Witnessing great team work • More patient contact with high degree of acuity

**Figure 1.**
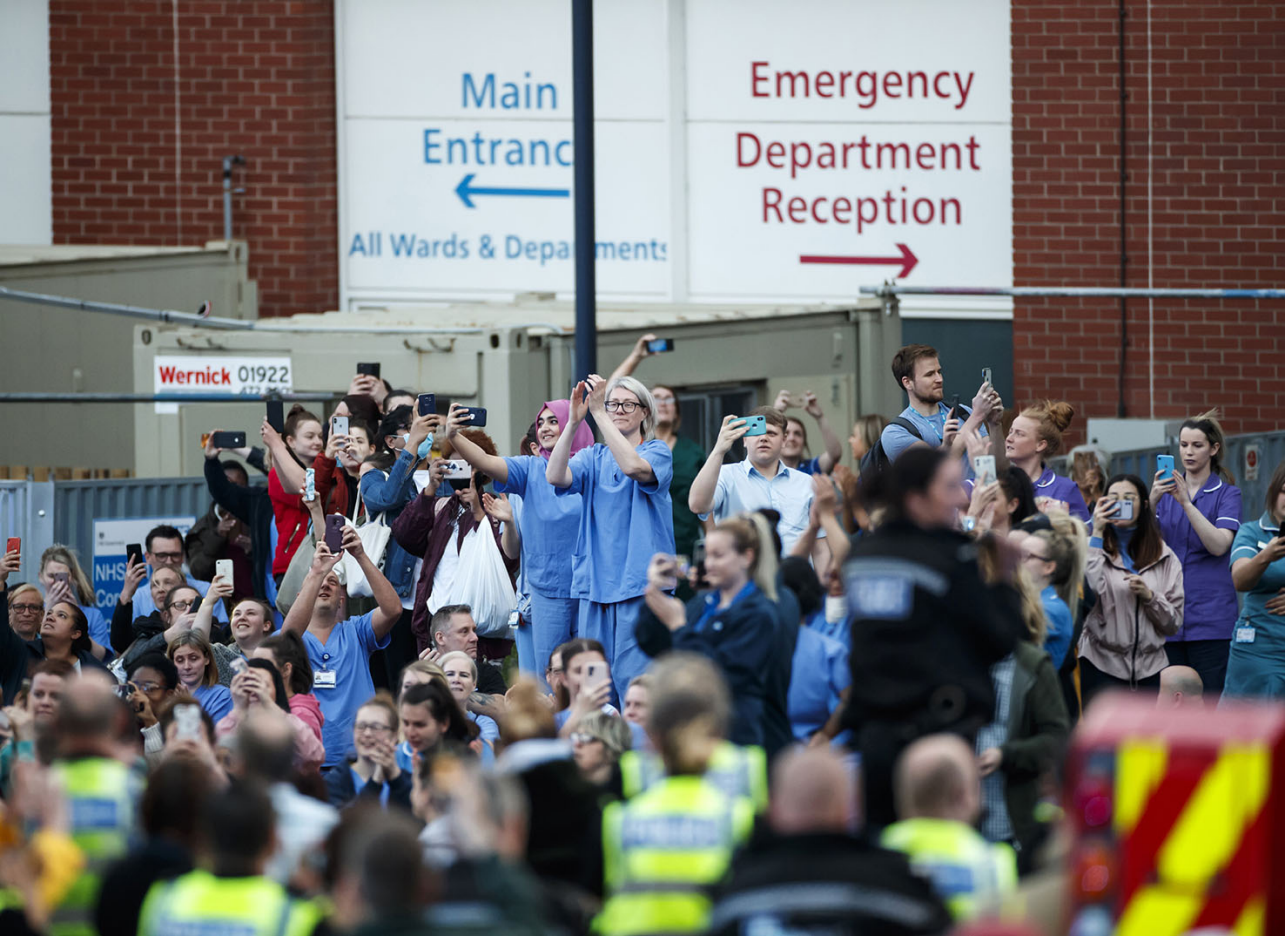
Members of the emergency services and the general public applaud NHS staff outside Leeds General Infirmary during Thursday’s nationwide applause for carers and the NHS. PA photos Ltd., with permission.

**Figure 2.**
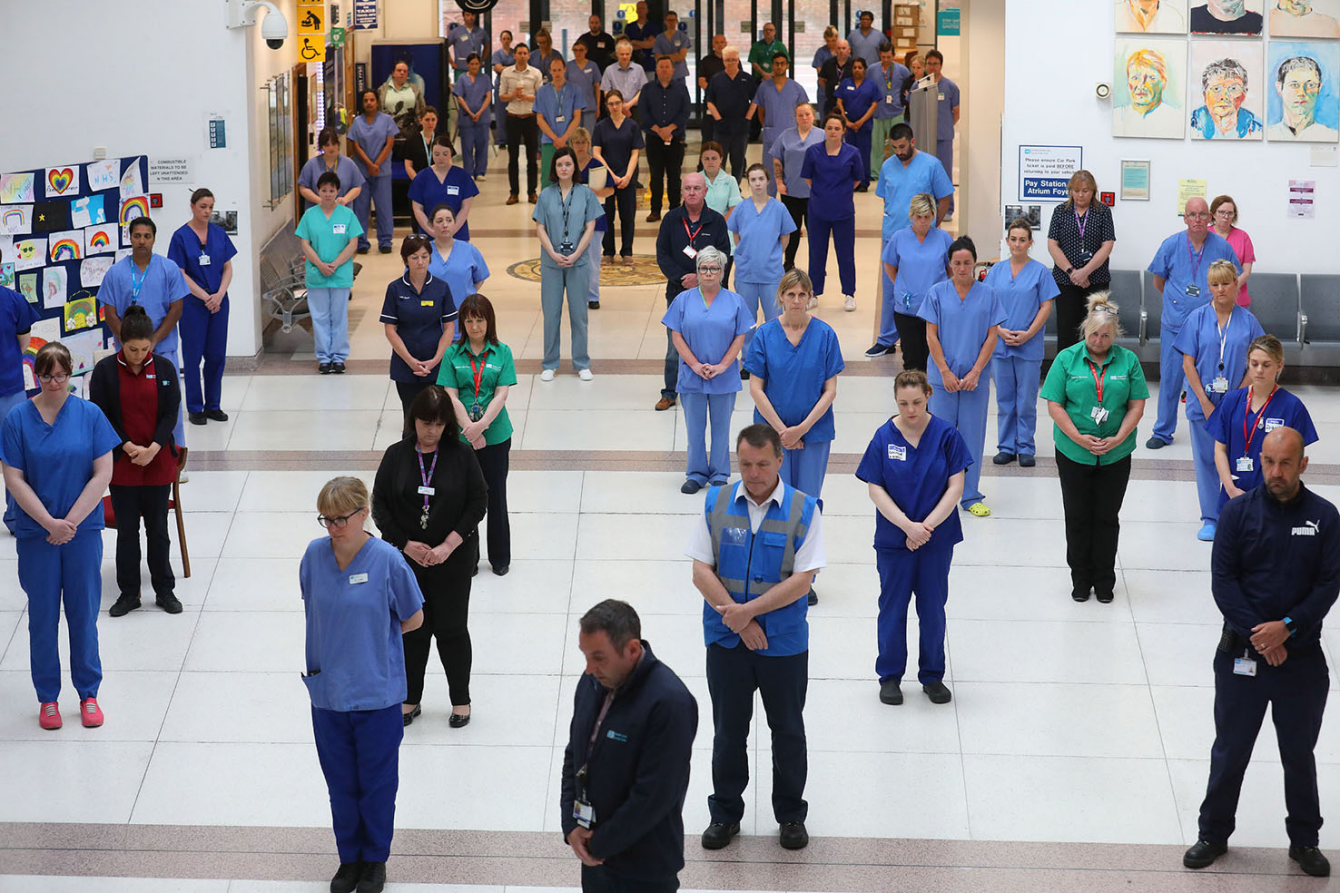
NHS staff at the Mater hospital in Belfast, UK, during a minute’s silence to pay tribute to the NHS on Tuesday 28.4.2020. PA photos Ltd., with permission.

Our recent experience as clinicians in the UK has prompted us to consider whether the pandemic may be even more transformative than discussed above. The experience of the public applause for NHS staff every Thursday evening ( Figure 1), as well as a recent minute of silence for healthcare providers who have died as a result of the pandemic ( Figure 2), have been unique, moving and powerful. We are convinced that such events must have profound effects on the generation of undergraduate learners witnessing it. Firstly, we think it is likely that young people will aspire to work in healthcare and that our profession may in the long run attract more vocational recruits. Secondly, we note the inter-professional camaraderie at work and also the fact that public support, appreciation and applause encompasses all members of the multi-disciplinary team. This leads us to believe that COVID-19 will also accelerate the drive for multi-professional learning. Finally, we believe that there are implications for curriculum design. Early research suggests that medical students would like to see more focus on public health education, crisis management technique, and also on mental health care ( Tabari, Amini and Moosavi, 2020). We agree and we would like to add uncertainty to the list. The need to teach “clinical” uncertainty has been emphasised before ( Birnie, 2020) but the current situation has been an opportunity for us to deal with a new level of uncertainty in our own daily work and we feel that we should teach our undergraduates how to deal with such challenges to prepare them for subsequent pandemics, and for their role in it.

## Conclusion

Four months after the World Health Organisation (WHO) first reported a cluster of patients with pneumonia in Wuhan, China, nobody can predict with certainty what the long‐term effects of COVID‐19 will be. We appreciate that the changes may not be quite as dramatic in countries less affected by the pandemic. However, in our own experience in the North West of England the pace of change has been astonishing and there can be no doubt that some of our local and regional learning environment will be radically transformed within months. Further unforeseen and unintended consequences beyond those outlined above are also possible. The concept of disruptive innovation stems from the corporate world and describes events that disrupt existing markets, value networks and structures and create a new normal. Previous pandemics have acted as disruptors for politics and society in general ( Snyder and Ravi, 2018) and COVID-19 will be no exception from this rule. As educators, we should perhaps begin to see the pandemic not solely as a threat to curriculum delivery and to our educational infrastructure but as a disruptor in the positive sense and as an opportunity. Health systems, clinical teams and individual clinicians are currently dealing with the ongoing pandemic and rightly so. However as educators we need to urgently consider the wider implications of this transformative change, discuss options to mitigate against the negative effects and harness positive aspects of the situation.

## Take Home Messages

• We speculate that the implications of the COVID-19 pandemic for educators will be profound.
• Previous pandemics acted as disruptors for society and COVID-19 will be no exception from this rule.
• As educators we need to urgently consider the implications of this transformative change, discuss options to mitigate against the negative effects and harness positive aspects of the situation.

## Notes On Contributors

**Alexander Woywodt** is a Consultant Nephrologist and Associate Undergraduate Dean at Lancashire Teaching Hospitals NHS Foundation Trust.

**Hetty Breed** is a third year medical student with the University of Manchester based at Lancashire Teaching Hospitals NHS Foundation Trust in Preston, UK.

**Colin Lumsden** is a Consultant Paediatrician and MbChB Programme lead at the University of Aberdeen, Aberdeen, UK.
