# Supplementary material for: COVID-19 – the ultimate disruptor?
Source: MedEdPublish (2016). 2020 May 20;9:104. [Version 1] doi: 10.15694/mep.2020.000104.1 (PMC10967686; doi:10.15694/mep.2020.000104.1)
Supplement: Peer review history

# Reviewer report: Reviewer response for version 1

**DOI:** 10.21956/mep.20068.r30825

**Published:** 2020-07-12

**Authors:** Olanrewaju Sorinola

**Affiliations:** 1 University of Warwick

This review has been migrated. The reviewer awarded 4 stars out of 5 Many thanks to the authors for a well written article. Even though it was an opinion piece there was enough background evidence to support most of the issues highlighted in the article. The authors considered both the immediate impact of Covid on education as well as the intermediate and ongoing issues that educators will be grasping with as time goes on. The impact of virtual clinics, telemedicine on students learning and clinical exposure were highlighted. The importance of using technology to support learning and make it work for learners is crucial. Learning outcomes based around online delivery, telemedicine, etc. now translates into educators needing to engage with various delivery platforms to enhance educational design and delivery. This is a change that is needed but educators will need support to achieve this in the new world post-Covid. However, the change is long overdue and we as educators and clinicians need to embrace this new ways of educating our students in both clinical and classroom scenarios.The authors gave examples which were from the UK but there is enough relevance for the international audience to appreciate the similarity to their own contexts. Every clinical educator understands the need to teach clinical uncertainty and as the authors rightly pointed out, the pandemic has been an opportunity for us to deal with a new level of uncertainty and the need to teach our undergraduates how to deal with such challenges. Finally the authors concluded that as educators, we should perhaps begin to see the pandemic not solely as a threat to curriculum delivery and to our educational infrastructure but as a disruptor in the positive sense and as an opportunity. While I agree with the authors’ statement, there is a lot of infrastructures that need to be provided quickly to make the rapid adaptations possible in the immediate period. At the same we still need to keep working on the transformative changes to sustain effective learning in the longer term. In summary, I think even though it is UK centric to some extent, it is still a very informative and valuable article which I would recommend.

Reviewer Expertise:

NA

No decision status is available

# Reviewer report: Reviewer response for version 1

**DOI:** 10.21956/mep.20068.r30826

**Published:** 2020-05-22

**Authors:** Barbara Jennings

**Affiliations:** 1 Norwich Medical School

This review has been migrated. The reviewer awarded 4 stars out of 5 Thank you to the authors for a very thoughtful and thought-provoking article that is well written and linked to some useful background literature.The short and long term challenges that covid-19 brought are well articulated. Competition for access to live patients by undergraduate & postgraduate trainees will be even greater if/as telemedicine flourishes - and it was tough enough in the face of expanding clinical education already.The title is apt. In medical schools and more widely, we have been observing or experiencing the struggle of introducing technology into healthcare delivery and clinical education. Learning objectives around telemedicine, and skills for information technology have been treated as fringe issues, and it has been hard to encourage most teachers to engage in the use of basic software and platforms to improve instructional design & delivery. But change has been needed for more than a decade. The pandemic-related lockdown has had a huge impact on practice that will no doubt lead to many permanent improvements in clinical & educational practice. The author’s description of increased camaraderie in health services during the acute crisis, and the impact this may have on multi-professional practice was particularly heartening. Queries: the citation linked to table 1 is unclear. Although this is an opinion piece, some data on student volunteers from a named institution were included in the analysis. I note in your ethics statement that the principles outlined in the Declaration of Helsinki were followed and I guess this relates to the data included? I enjoyed reading this article and will recommend it to colleagues who deliver undergraduate and postgraduate medical and clinical education.

Reviewer Expertise:

NA

No decision status is available

# Reviewer report: Reviewer response for version 1

**DOI:** 10.21956/mep.20068.r30823

**Published:** 2020-05-21

**Authors:** Maria de los Angeles Fernandez-Altuna

**Affiliations:** 1 Universidad Nacional Autonoma de Mexico Facultad de Medicina

This review has been migrated. The reviewer awarded 4 stars out of 5 Thank you for writting this article from the point of view of educational practice during the COVID-19 pandemic. I liked very much the broad approach taking into account not only what happened to medical care, but also to medical education, opportunities (like telemedicine) and some negative aspects (like medical students loosing practice and lack of preparation).Telemedicine in the medical specialties has already changed with this pandemic. I liked how you portrait that change in your country.In order to improve esthetics of the manuscript, I would respectfully advise to give more space between each parragraph, it would be easier to read.

Reviewer Expertise:

NA

No decision status is available

# Reviewer report: Reviewer response for version 1

**DOI:** 10.21956/mep.20068.r30824

**Published:** 2020-05-20

**Authors:** P Ravi Shankar

**Affiliations:** 1 American International Medical University

This review has been migrated. The reviewer awarded 4 stars out of 5 This article dealing with the COVID-19 crisis takes a broader perspective at the changes in medicine and medical education due to the crisis. The impact of telemedicine and of consultations being held online on medical student learning opportunities has been mentioned. The convenience of telemedicine for both patients and practitioners may result in becoming more common. As the authors mention student learning opportunities should be built into the telemedicine consultation. The impact of enhanced spending during the pandemic and of subsequent austerity has been mentioned. There may be significant deficiencies in students’ clinical skills despite an attempt to address some of these through online learning. Hands on learning may help address some of these after the situation improves. Some of the terms especially in table 1 are more related to the United Kingdom context and may need some explaining for international readers. It will be interesting to note possible descriptions of the influenza pandemic’s impact on medical education during the early twentieth century. The importance of public health has been underlined and more medical students may be interested in taking up careers in public health. There have been areas which have been traditionally neglected like public health, infectious diseases and antimicrobials and these may receive more attention and funding hopefully.

Reviewer Expertise:

NA

No decision status is available
